# Behavioural alterations are independent of sickness behaviour in chronic experimental Chagas disease

**DOI:** 10.1590/0074-02760150300

**Published:** 2015-12

**Authors:** Glaucia Vilar-Pereira, Leonardo Alexandre de Souza Ruivo, Joseli Lannes-Vieira

**Affiliations:** Fundação Oswaldo Cruz, Instituto Oswaldo Cruz, Laboratório de Biologia das Interações, Rio de Janeiro, RJ, Brasil

**Keywords:** Chagas disease, Trypanosoma cruzi, behavioural alterations, sickness behaviour, depression, anxiety

## Abstract

The existence of the nervous form of Chagas disease is a matter of discussion since Carlos Chagas described neurological disorders, learning and behavioural alterations in *Trypanosoma cruzi*-infected individuals. In most patients, the clinical manifestations of the acute phase, including neurological abnormalities, resolve spontaneously without apparent consequence in the chronic phase of infection. However, chronic Chagas disease patients have behavioural changes such as psychomotor alterations, attention and memory deficits, and depression. In the present study, we tested whether or not behavioural alterations are reproducible in experimental models. We show that C57BL/6 mice chronically infected with the Colombian strain of *T. cruzi* (150 days post-infection) exhibit behavioural changes as (i) depression in the tail suspension and forced swim tests, (ii) anxiety analysed by elevated plus maze and open field test sand and (iii) motor coordination in the rotarod test. These alterations are neither associated with neuromuscular disorders assessed by the grip strength test nor with sickness behaviour analysed by temperature variation sand weight loss. Therefore, chronically *T. cruzi*-infected mice replicate behavioural alterations (depression and anxiety) detected in Chagas disease patients opening an opportunity to study the interconnection and the physiopathology of these two biological processes in an infectious scenario.

The haemoflagellate protozoan *Trypanosoma cruzi* causes American trypanosomiasis or Chagas disease, which afflicts approximately six-seven million people worldwide (WHO 2015). The existence of a nervous form of Chagas disease is matter of debate since the description by Carlos Chagas of neurological and behavioural changes in chronically infected patients (Chagas 1911). Although this work was highly criticised because of the lack of appropriated controls, in the last decades several studies supported the existence of behavioural alterations in chronic Chagas disease patients [reviewed by Silva et al. (2010)]. Further, more than hundred years after the discovery, little is known about the pathophysiology of the behavioural changes in Chagas disease. In most patients, the clinical manifestations of the acute phase, including neurological disorders, spontaneously resolve without apparent sequel in the chronic phase of infection (Prata 2001, Pittella 2009). Nevertheless, patients with chronic Chagas disease exhibit many neuropathies such as debilitation of muscle tendon reflex, myoclonic seizures, marching disorders, bradykinesia, and paresis (Jörg & Rovira 1981). The presence of symptoms of Chagas disease is a risk factor for the development of feelings of hopelessness and emotional difficulties. Patients upon learning of Chagas infection develop reactive symptoms ranging from anxiety, depression to alexithymia syndrome (Jörg & Rovira 1981). Patients with the symptomatic form of Chagas disease have more psychological and physical symptoms of stress and anxiety, and lower capacity of resilience compared with asymptomatic individuals (Mota et al. 2006). Considering the chronic nature of Chagas disease, in persistence of stress the patients may develop depressive symptoms which may have a negative impact on functional performance and quality of life (Hueb & Loureiro 2005, Ozaki et al. 2011, Guimarães et al. 2014).

Previously, we have shown that depressive-like behaviour in the chronic phase of experimental *T. cruzi* infection in mice models is independent of the prior existence of acute meningoencephalitis, and, therefore, is not a sequel of this (Vilar-Pereira et al. 2012). However, it has not been explored whether or not anxiety is present in the chronic phase of experimental Chagas disease in association with depressive behaviour. Anxiety and depression are terms used to describe emotional states associated with stressful events or psychological difficulties in pathological conditions (Putilina 2014). These behavioural changes often develop in comorbidity and many of their symptoms are similar. Further, depression is common in patients with anxiety, and anxiety is frequently reported in depressed patients, both poor prognosis predictors (Nutt 2002, Nutt et al. 2006).

Mice models reproduce aspects of anxiety (Steiner et al. 2011, Greene-Schloesser et al. 2011). However, in animal models sickness behaviour may contribute to behavioural changes (Rogers et al. 2001, Maes et al. 2012). Sickness behaviour causes disturbs as fever, loss of libido, decreased locomotor activity, loss of appetite, disinterest in the social and physical environment, and general anhedonia (Rogers et al. 2001, Dantzer et al. 2008, Maes et al. 2012). Acutely*T. cruzi*-infected mice show signs of sickness behaviour as reduced exploratory and motor activity, decreased consumption of water and chow, weight loss, and temperature variations (da Silva et al. 2012). Importantly, some authors regard clinical anxiety and depression as a form of sickness behaviour and/or as a consequence of the sickness behavioural response (Dantzer et al. 2008, Maes et al. 2012).

In this work, we hypothesised that the behavioural abnormalities described in patients with Chagas disease depression and anxiety are nonpsychological features, but a neurochemical process resulted of the parasite/host interactions and, therefore, possible of being reproduced in a model of chronic experimental *T. cruzi* infection. Further, we assessed whether there is or not a relation between depression and anxiety with loss of muscle strength and/or sickness behaviour in the chronic phase of experimental Chagas disease.

## MATERIALS AND METHODS

*Mouse model of chronic infection by T. cruzi* - Five-seven-week-old female C57BL/6 (H-2^b^) mice obtained from the animal facilities (Laboratory Animals Breeding Center) of the Oswaldo Cruz Foundation (Fiocruz), Rio de Janeiro, Brazil, were maintained under specific pathogen free, standard conditions (with temperature and relative humidity of approximately 22 ± 2°C and 55 ± 10%, respectively) and received food and water *ad libitum*. The noninfected (NI) control experimental group consisted of four-five mice per experiment (14 mice) and infected experimental group consisted of eight-18 mice per experiment (40 mice).

*Experimental infection* - Mice were infected with the Colombian*T. cruzi* Type I strain (Zingales et al. 2009), which is considered myotropic (Melo & Brener 1978) and has previously been shown to colonise the central nervous system (CNS) (Silva et al. 1999, 2015, Roffê et al. 2003). The Colombian strain is maintained by serial passages from mouse to mouse every 35-45 day in the Laboratory of Biology of the Interactions, Oswaldo Cruz Institute (IOC), Fiocruz. For the present study, C57BL/6 mice were infected intraperitoneally with the low inoculum of 100 blood trypomastigote (bt) forms suspended in 0.2 mL of sterile buffered saline. Parasitaemia was estimated using 5 μL of blood obtained from the tail vein. The first circulating parasites were detected in the blood at 14 days post-infection (dpi), marking the onset of the acute phase of infection. The peak of parasitaemia was observed between 42-45 dpi and trypomastigotes were rarely found in the blood at 90 dpi, characterising the onset of the chronic phase of infection, as previously described (Silva et al. 1999, Vilar-Pereira et al. 2012).

*Tail-suspension test (TST)* - Mice subjected to the short-term inescapable stress of being suspended by their tail will develop an immobile posture. Immobility is defined as the absence of initiated movements and includes passive swaying. In this test, adapted from Steru et al. (1985), the mouse was hung upside-down using adhesive tape to fix its tail to a vertical surface (an iron rod with a height of 30 cm). A white platform was positioned horizontally 20 cm below the iron rod, just under the mouse’s forepaws, in such a way that the mouse could lightly touch the platform and minimise the weight sustained by its tail until the recording began. The animal’s behaviour was recorded with a video camera for 5 min (Sony, USA). The total time of immobility was measured. The mouse was considered immobile when it was not struggling, attempting to catch the adhesive tape, or showing body torsion or jerky movements.

*Forced swim test (FST)* - This procedure consisted of placing the mouse inside a cylindrical glass tank (height 35 cm, diameter 25 cm) containing clean water at 24-26ºC to a level of 20 cm above the bottom (Porsolt et al. 1977). The animal was left in the cylinder for 6 min. After the first 2 min of habituation, the total duration of immobility was measured over a period of 4 min of test. The mouse was considered to be immobile when it remained floating passively in the water or was making slight movements to keep its head above the water. A video camera (Sony) was placed on top of the water tank to record the test. The water was changed before the introduction of each animal. The animal was dried with gauze and replaced in its cage.

*Marble burying test (MBT)* - This assay was described in mice (Njung’e & Handley 1991), as these animals exhibit burying behaviour in the presence of aversive stimuli. Mice were placed individually in transparent acrylic box containing 20 glass beads distributed over the sawdust. After the 30 min period, each mouse was removed from the box. Subsequently, the number of buried glass beads in sawdust was registered. After testing each animal, the exchange of sawdust was performed and the glass beads were cleaned with a solution of 70% alcohol and dried with towel paper.

*Open-field test (OFT)* - Emotionality, locomotor, and exploratory activities were tested using a modified version of the open field arena; because the animals have never been in the test environment, they tend to explore it (Hall 1941). The open field was a white acrylic arena measuring 60 cm x 60 cm. The floor of the apparatus was divided by black grid lines into 49 squares of approximately 8.5 cm each and two imaginary areas - the periphery (40 squares along the walls) and centre (9 squares in the central area of the apparatus). Each mouse was tested in 5-min sessions. Activities were recorded using a video camera (Sony). To assess the number of behavioural elements, the following parameters were utilised: (i) total locomotor activity, i.e., when the animals crossed each grid line with all four paws in the total area, (ii) inner locomotor activity, i.e., when the animals crossed each grid line with all four paws in the central area. The apparatus was cleaned with 70% alcohol and dried with gauze between tests.

*Elevated plus maze test (EPMT)* - This assay evaluates anxiety-like behaviour (Lister 1987). The EPMT consisted of two open arms each (50 × 10 cm) crossed with two closed arms with 40 cm high walls. In this test, a mouse was selected randomly and placed in the centre zone, facing the corner between a closed arm and an open arm. Its behaviour was recorded for 5 min using a digital video camera (Sony) mounted above the maze. The behavioural parameters related to anxiety were the number of entries and time spent in the open arms. An arm entry was scored when all four paws of the animal were placed in an arm. Before the next mouse was introduced, the maze was cleaned with a solution of 70% alcohol and dried to eliminate the odour and trace of the previously tested mouse.

*Rotarod (RRT)* - This test is used to assess motor coordination and balance. An automated device of RRT was used (EFF 411; Insight, Brazil), which consisted of a plexiglass box with a cylinder of 8 cm in diameter, located approximately 20 cm across the machine from the ground, kept in rotation by a motor. The box was divided into four bays, approximately 10 cm wide, allowing the analysis of four mice simultaneously. For the test, each mouse was placed on the drum already in motion at the initial speed of 10 rpm. Results are expressed as average latency on RRT. In the fall, the timer which checked the equilibrium time is automatically stopped; the animals are brought back to their stall and the timer reactivated, accounting for the total falls after 5 min at a maximum speed of 37 rpm. The time of mice permanency in the apparatus was recorded in a maximum time limit of 300 s. For habituation/training, mice were placed for walking on the rotary drum at minimum speed (10 rpm) for 5 min the day before the test day.

*Grip strength meter test (GSMT)* -This is a noninvasive method widely used to assess the strength of the members of mice and may disclose a neuromuscular disorder (van Riezen & Boersma 1969). The muscle strength was evaluated through the grip strength meter (EFF 305; Insight). The GSMT is based on the natural tendency of the mouse to grab a horizontal metal bar when slightly pulled by the tail for 2-3 s. The bar is attached to a force transducer which measures the traction force peak (in grams) that is displayed on a digital screen.

*Standardised conditions to perform behavioural tests* - All behavioural tests occurred during the light phase between 08:00 am-05:00 pm and were recorded with a DSC-DVD810 video camera (Sony). To minimise stress and maximise familiarity, all behavioural tests applied to the different experimental groups were conducted in an environment with a 12-h light and 12-h dark cycle, a room temperature of 22 ± 2°C and an ambient noise level of approximately 40 dB produced by an air conditioner. Mice were subjected to behavioural tests at 150 dpi. The animals were subjected to the following sequence of tests: OFT, GSMT, EPMT, MBT, RRT, TST, and FST. No animal was re-tested. To further assess sickness behaviour that might have contributed to behavioural alterations (Rogers et al. 2001), we checked temperature with a rectal probe (Thermometer DT-610B; ATP, USA), apathy, and body weight loss. Deaths were registered weekly.

*Statistical analysis* - Data are expressed as arithmetic means ± standard deviation. Student’s *t*test was used to analyse the statistical significance of the observed differences. The Kaplan-Meier method was used to compare the survival curves of the studied groups. The statistical tests were performed with GraphPad Prism. Differences were considered statistically significant when p < 0.05.

*Ethics -* This study was carried out in strict accordance with the recommendations in the Guide for the Care and Use of Laboratory Animals of the Brazilian National Council of Animal Experimentation (cobea.org.br) and the federal law 11.794 (8 October 2008). The Ethical Commission on Animal Use of Fiocruz (licenses 004/09 and LW10/14) approved all the procedures used in this study. All presented data were obtained from three (D4, D1G, D2G) independent experiments (Experiment Register Books 49, 53, and 57, LBI/IOC-Fiocruz).

## RESULTS

**Fig. 1:**
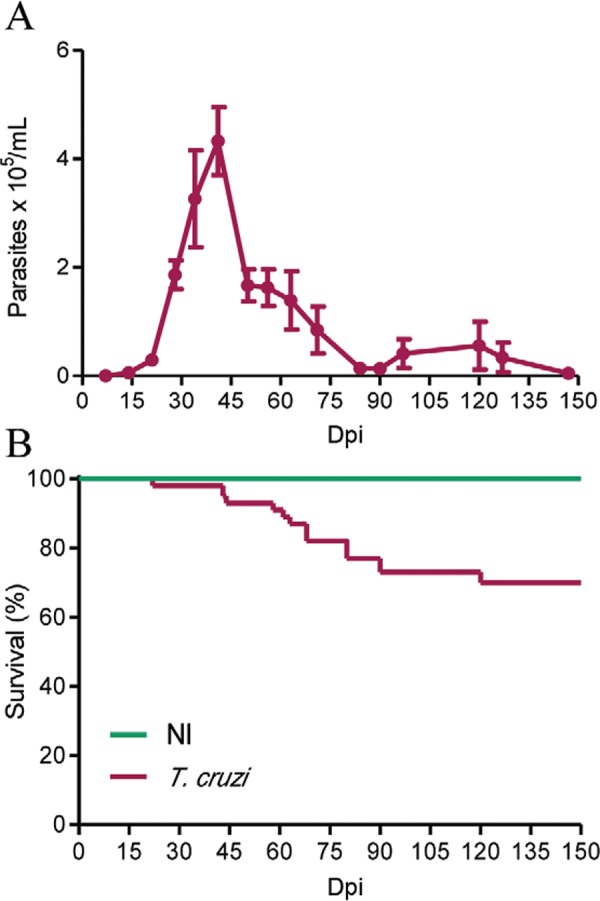
: parasitaemia curve and mortality during experimental infection of C57BL/6 mice by *Trypanosoma cruzi*. Mice were infected with 100 trypomastigotes of the Colombian strain of *T. cruzi* (A). Survival curve, compared with noninfected (NI) matched for age and sex, shows that ~70% of infected animals survived the acute phase and developed chronic infection (B). The groups were composed of 10-18 mice. Data are representative of three independent experiments. dpi: days post-infection.

*Model of chronic experimental Chagas disease* - Initially, we established a murine model that reproduces aspects of chronic Chagas disease. To this end, C57BL/6 mice were infected with the low inoculum of 100 bts of the Colombian *T. cruzi* Type I strain. The parasitaemia peak occurred at 42-45 dpi and trypomastigotes were rarely found in the blood at 150 dpi (Fig. 1A). Most of the infected mice survived the acute phase (26/40 mice in 3 independent experiments), without trypanocidal treatment, and developed a chronic infection (Fig. 1B), as formerly described (Talvani et al. 2000, Silverio et al. 2012, Pereira et al. 2014a). In a previous work, we showed that C57BL/6 mice are resistant to CNS inflammation induced by chronic infection with the Colombian strain (Roffê et al. 2003). Therefore, this model reproduces important aspects of the chronic phase of Chagas disease and allowed us to test whether or not the Colombian-infected mice present the behavioural alterations depression and anxiety and, if so, whether these changes were associated with sickness behaviour.

**Fig. 2:**
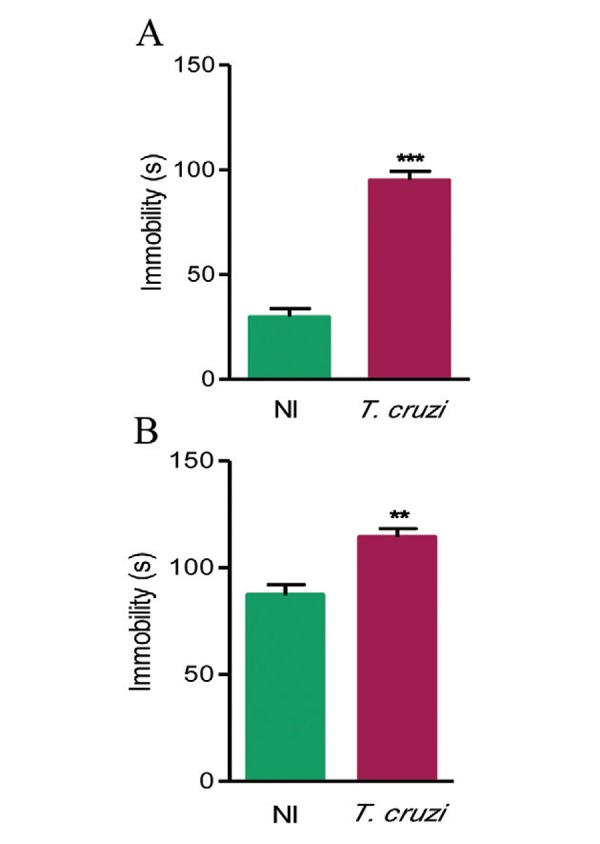
: depressive-like behaviour in C57BL/6 mice in the chronic phase (150 days post-infection) of infection with *Trypanosoma cruzi*. Mice were infected with 100 trypomastigote forms of the Colombian strain and subjected to the tail-suspension test (A) and forced swim test (B). Quantification of immobility time was observed in the period of 4 min. Data represent means ± standard deviation. The groups were composed of four-18 mice. Data are representative of three independent experiments. NI: noninfected; **: p < 0.01; ***: p < 0.001.

*Depressive-like behaviour is present in chronic experimental T. cruzi infection* - To assess depressive-like behaviour we used the FST and TST. At 150 dpi, there was a significant increase in the immobility time in TST (p < 0.001) (Fig. 2A) and FST (p < 0.01) (Fig. 2B) of the Colombian-infected mice when compared with sex and age-matched NI controls.

**Fig. 3:**
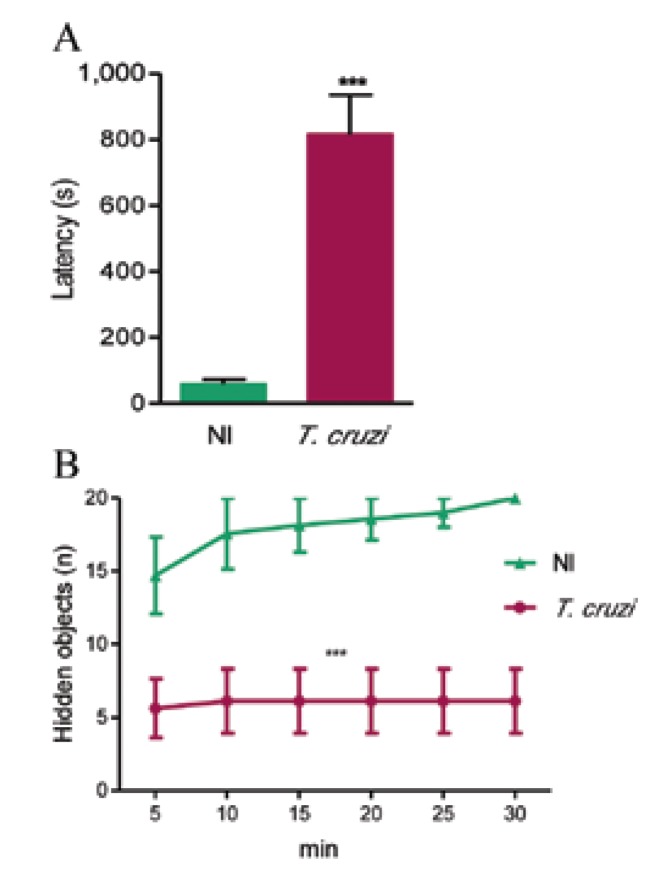
: anxiety behaviour in C57BL/6 mice during infection with*Try- panosoma cruzi*. Animals were infected with 100 trypomastigotes of the Colombian strain and subjected to the marble burying test. The latency and the quantification of the number of hidden balls were analysed in a 30 min period. Data represent means ± standard deviation. The groups were composed of seven-16 mice. Data are representative of three independent experiments. Asterisks mean p < 0.001. NI: noninfected.

**Fig. 4:**
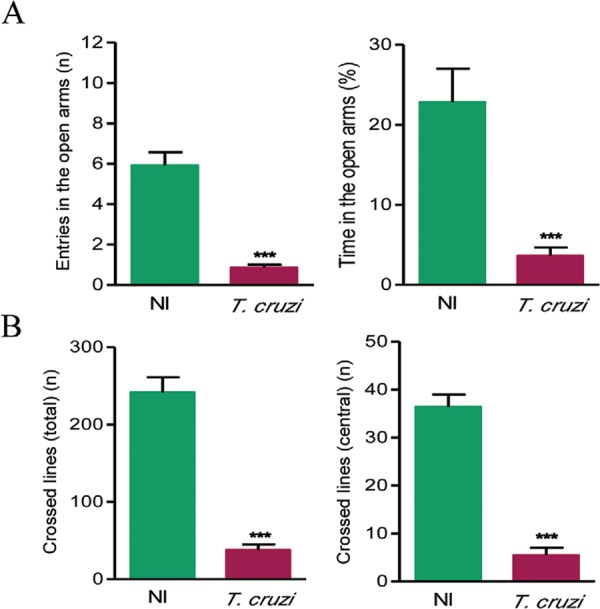
: anxiety and locomotor/exploratory abnormalities in*Trypanosoma cruzi*-infected C57BL/6 mice. Mice were infected with 100 trypomastigotes of the Colombian strain and subjected to the test the elevated plus maze. Sex and age-matched noninfected (NI) controls were analysed in parallel. A: anxiety behaviour in the chronic phase of infection [150 days post-infection (dpi)]. The graphs show the number of entries and time spent in the open arms in 5-min sessions; B: locomotor/exploratory activity. NI and *T. cruzi*-infected mice were subjected to the open field test for 50-min sessions. The graphs show the number of total and central lines crossed in the chronic (150 dpi) phases of infection. Data are expressed as means ± standard deviation. The groups consisted of 12-18 infected mice per test. This figure is representative of three independent experiments. Asterisks mean p < 0.001.

*Anxiety-like behaviour and locomotor/exploratory alterations are present in chronically T. cruzi*-*infected C57BL/6 mice* - To test whether repetitive/compulsive behaviour is present in the chronic phase of experimental *T. cruzi* infection, we subjected mice to the MBT and analysed the latency and the number of hidden balls. Chronically Colombian-infected mice presented a significant increase in time of latency to approach and push sawdust toward a marble (Fig. 3A). Further, these mice showed a significant decrease in quantification of the number of hidden balls (p < 0.001) in 30-min sessions when compared to NI controls (Fig. 3B). Therefore, chronically infected mice did not show the innate repetitive/compulsive behaviour when exposed to glass beads. To evaluate anxiety, we used the EPMT with analysis of the frequency of entries and the time the mouse spent in the open arms in 5-min sessions. Our data support that chronically *T. cruzi*-infected mice show reduced numbers of entries in the open arms of the apparatus as well as remained significantly reduced time in the open arms (p < 0.001) in comparison with NI controls (Fig. 4A); therefore, these data support the existence of anxiety-like behaviour in chronically *T. cruzi*-infected mice.

To test whether behavioural alterations of locomotor/exploratory pattern are present in *T. cruzi*-infected mice, they were subjected to the OFT and the numbers of total and central crossed lines were analysed. Chronically Colombian-infected C57BL/6 mice exhibited a significant decrease in the locomotor/exploratory activities in 5-min sessions expressed as reduced numbers of crossed total and central lines (p < 0.001) when compared with NI controls (Fig. 4B).

**Fig. 5:**
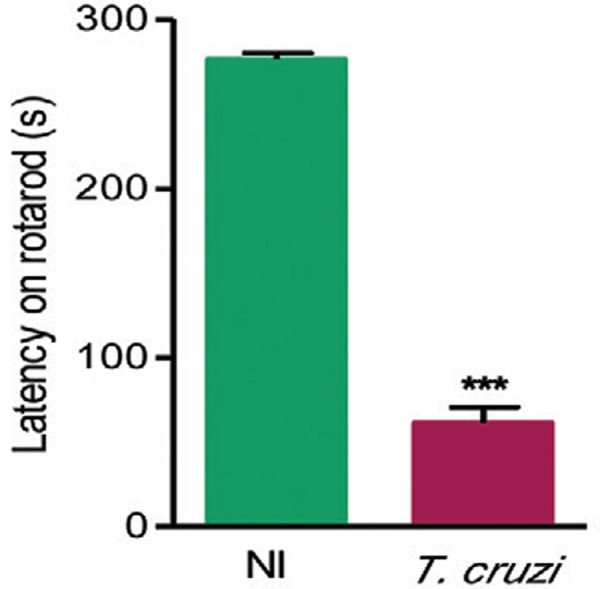
: motor coordination assay in *Trypanosoma cruzi*-infected C57BL/6 mice. Mice were infected with 100 trypomastigotes of the Colombian strain and subjected to the rotarod test, at 150 days post-infection. Motor coordination was analysed in*T. cruzi*-infected and noninfected (NI) controls. Data are expressed as means ± standard deviation. The experimental groups consisted of five-eight mice per group. Representative figure of three independent experiments. Asterisks mean p < 0.001.

*Motor coordination is reduced in chronic experimental T. cruzi infection* - When evaluated by the RRT, chronically *T. cruzi*-infected mice showed impaired performance and decrease on motor coordination revealed by significantly (p < 0.001) reduced time to fall from the spinning wheel when compared with NI controls (Fig. 5).

**Fig. 6:**
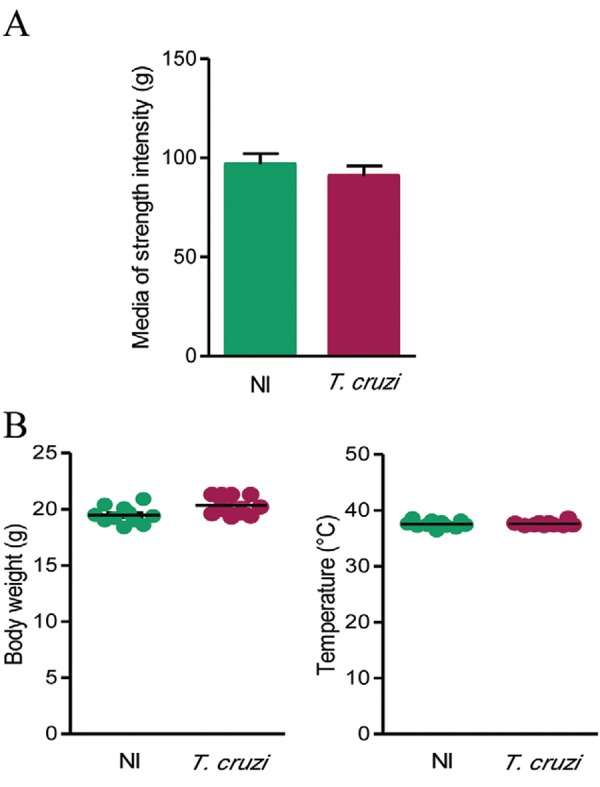
: analysis strength and sickness behaviour in C57BL/6 mice chronically infected with *Trypanosoma cruzi*. Mice were infected with 100 trypomastigotes of the Colombian strain and subjected to the grip strength muscle (GSM) and a checked for body weight and temperature at days post-infection. A: the graph shows the GSM results of infected mice compared with sex and age-matched noninfected (NI) controls; B: body weight (g) and rectal temperature (°C) were registered in infected mice compared with NI controls. Data are expressed as means ± standard deviation. The experimental groups consisted of five-12 mice per group. Representative figure of three independent experiments.

*Chronically T. cruzi-infected C57BL/6 mice do not show loss of muscle strength* - To determine if the infection with *T. cruzi*is accompanied by a decrease in muscular strength, we performed GSMT. Interestingly, there was no significant difference (p > 0.05) in grip strength when chronically infected mice were compared with their NI matched controls (Fig. 6A).

*Absence of sickness behaviour in chronic experimental Chagas disease in C57BL/6 mice* - Considering that sickness features may contribute to behavioural alterations such as decreases in spontaneous locomotor/exploratory activities (Rogers et al. 2001), we further assessed sickness behaviour by checking body weight loss (which reveals loss of appetite), apathy and increase in temperature (indicative of fever). At 150 dpi, the clinical observation showed that apathy and prostration were not detected in infected mice. Further, chronically infected mice showed neither body weight loss nor alteration in rectal temperature when compared with sex and age-matched NI controls (Fig. 6B). Altogether, these data demonstrate that C57BL/6 mice infected with a low inoculum of the Colombian strain do not exhibit signs of sickness behaviour.

## DISCUSSION

In the present study we challenged the hypothesis that the behavioural abnormalities depression and anxiety described in Chagas disease patients are nonpsychological features and could be reproduced in a model of experimental *T. cruzi* infection. Therefore, we tested whether infection with a*T. cruzi* Type I strain that allows survival and development of the chronic phase of infection led to the behavioural alterations depression, anxiety, and locomotor/exploratory disorders. Our results show that chronic infection of C57BL/6 mice with the Colombian strain reproduces these behavioural changes. Importantly, in this model of chronic experimental Chagas disease the behavioural alterations are independent of the loss of muscle strength and sickness behaviour.

The infection of C57BL/6 mice with the low inoculum of the Colombian *T. cruzi* Type I strain resulted in high parasitaemia that dramatically dropped in the chronic phase of infection. In the three independent experiments, the majority of the infected mice survived and developed chronic infection, corroborating previous findings (Silva et al. 1999, 2015, Roffê et al. 2003, Silverio et al. 2012, Vilar-Pereira et al. 2012). Thus, this model was considered suitable to investigate possible behavioural changes during the chronic phase of infection.

The involvement of the CNS in Chagas disease is recognised in the acute phase of the infection, when meningoencephalitis is described as a significant cause of death (Prata 2001, Rassi et al. 2010). However, there is no consensus on the existence of a defined clinical nervous form in the chronic phase of Chagas disease, especially due to the lack of an anatomical basis that can characterise it (Pittella 2009). The knowledge of being a Chagas disease carrier can elicit psychological disturbances, particularly because there is no cure for this disease (Mota et al. 2006). As a result of the disease and/or the difficulties that are associated as they do not know how to confront this condition, the carriers of Chagas disease may develop depressive symptoms. Therefore, Chagas disease patients need to face physical, psychological, social, and economic difficulties, which can compromise their quality of life (Hueb & Loureiro 2005, Osaki et al. 2011). There is a gap in the knowledge on the association of chronic Chagas disease and the comorbidities depression and anxiety and their impact on the clinical picture and disease prognosis. Thus, experimental models that reproduce aspects of the behavioural changes (depression and anxiety) detected in Chagas disease patients may contribute to the understanding of physiopathological factors leading these behavioural alterations.

In the present study, we used the TST and FST to assess depressive-like behaviour, as described in mouse models of chronic stress (Ma et al. 2011), immune challenge with LPS (Painsipp et al. 2011), and experimental Chagas disease (Vilar-Pereira et al. 2012). Depressive-like behaviour was detected in the Colombian-infected C57BL/6 mice when a significant increase in immobility was detected during the late phase of infection (150 dpi). Thus, these data support the existence of a nonpsychological component of this disorder in this model of experimental Chagas disease, corroborating previous findings (Vilar-Pereira et al. 2012). Thus, this model is proper to test the coexistence of the other behavioural abnormality anxiety and locomotor/exploratory disorders in the chronic *T. cruzi*infection.

Anxiety is considered a psychiatric disorder characterised by disproportional phobia in situations that represent stress, danger, real threats, or daily challenges (Moutier & Stein 1999). Diagnosis of seropositivity for Chagas disease is received with anxiety, fear, apprehension, or despair (Uchoa et al. 2002), and Chagas disease patients show high levels of psychological and physical symptoms of stress (Mota et al. 2006). In mouse models, the innate repetitive/compulsive behaviour assessed by the MBT was previously associated to anxiety (Borsini et al. 2002, Li et al. 2006). In our study, after being exposed to glass beads, which are supposed to represent an extraneous object whose presence can be a threat, NI control mice exhibited intense activity to hide balls. Conversely, chronically infected mice did not show such behaviour. These data support that our *T. cruzi*-infected mice did not present the innate repetitive/compulsive behaviour. The most widely used tests to assess anxiety in experimental models are EPMT and OFT (Hall 1941, Lister 1987). In the EPMT, mice will explore all parts of the maze (open and closed arms). However, under anxiogenic condition mice will avoid open and high places (Morato & Brandão 1997). Our data support that chronically infected C57BL/6 mice present a significant reduction in the number of entries in the open arms. Altogether, these results suggest that mice chronically infected with the Colombian *T. cruzi* strain did not show the repetitive/compulsive behaviour but present anxiety-like behaviour. Indeed, previous report support that repetitive/compulsive behaviour and anxiety are dissociated behaviour alterations (Greene-Schloesser et al. 2011). In the OFT, ambulation as spontaneous behaviour may be the result of exploratory and emotional impulses. However, there is a conflict between exploring the new environment, which is the natural tendency of mice, and the anxiety and fear generated by the novelty to confront new situations and environments (Arakawa & Ikeda 1991, Rex et al. 1996). The investigation of ambulation activities showed that chronically Colombian-infected mice exhibit a decrease in locomotor/exploratory activities when compared with age and sex-matched NI controls. In contrast, motor alterations were absent in mice that survived the acute infection with the Tulahuen *T. cruzi* Type VI strain (Caradonna & Pereiraperrin 2009). Further, mice chronically infected with the Colombian Type I strain, but not with Y*T. cruzi* Type II strain, present depressive-like behaviour (Vilar-Pereira et al. 2012). Therefore, the association of behavioural changes with infection by specific *T. cruzi* type strain(s) need to be explored in experimental models and Chagas disease patients. Importantly, in chronically Colombian-infected C57BL/6 mice, despite the low parasitism and absence of inflammatory infiltrates in the CNS (Silva et al. 1999, 2015, 2015, Roffê et al. 2003), there are remarkable behavioural changes, consistent with signs of depression and anxiety. The mechanisms that lead to this framework require further studies to be clarified.

Considering that sickness features may contribute to behavioural alterations such as decreases in spontaneous locomotor/exploratory activity (Rogers et al. 2001), we assessed sickness behaviour by checking apathy, body weight loss (which reveals loss of appetite), and temperature (since increase in temperature indicates fever). Apathy and prostration were not detected in chronically Colombian-infected C57BL/6 mice. Thus, these data ruled out the presence of sickness behaviour and reinforced the existence of the morbidities depression and anxiety in the model of chronic infection of C57BL/6 mice with the Colombian strain. Dissociated as possible interference related with sickness behaviour, our model reflects important aspects to form bridges between animal models of neurological and neuropsychiatric disorders observed in humans. Therefore, this model of chronic experimental Chagas disease may offer an opportunity to unravel the cellular and molecular mechanism triggering these behavioural abnormalities that may contribute to decrease the quality of life of Chagas disease patients.

In the last decades, the extension of life time had as consequence prevalence of more than 10% of depressive and anxiety disorders (Belmaker & Agam 2008). Besides facing considerable disruption to their psychological well-being, individuals suffering from these disorders are also at considerably greater risk for additional conditions as heart disease and obesity (Sheps & Sheffield 2001, Rumsfeld & Ho 2005). Depression and other behavioural alterations have been proposed as secondary consequences of the chronic inflammatory chagasic cardiomyopathy (Mosovich et al. 2008). Indeed, chronically Colombian-infected C57BL/6 mice also present electrical and functional heart alterations (Silverio et al. 2012, Pereira et al. 2014a, b, 2015). These cardiac abnormalities are responsive to the immune modulators anti-tumour necrosis factor (Pereira et al. 2014b) and pentoxifylline (Pereira et al. 2015), therapies that also hampered depressive-like behaviour (Vilar-Pereira et al. 2012). Therefore, in chronically infected mice the cardiac and behavioural abnormalities may be related to the systemic inflammatory profile. Altogether, our model of the comorbidities depression and anxiety in the presence of severe cardiomyopathy may offer an opportunity to achieve mechanistic insights into the complexity of *T. cruzi* infection. More broadly, this model is suitable to explore therapeutic tools, which may also be applicable to noninfectious disorders.
